# Generalized Mechanism Model for Ecosystem Hysteresis

**DOI:** 10.1002/advs.202509008

**Published:** 2026-01-14

**Authors:** Yanbin Hao, Xin Wang, Jie Liu, Mingzi Wu, Jianqing Du, Kai Xue, Xiaoning Song, Xiaoyong Cui, Tong Zhao, Yanfen Wang

**Affiliations:** ^1^ Beijing Yanshan Earth Critical Zone National Research Station University of Chinese Academy of Sciences Beijing China; ^2^ College of Life Sciences University of Chinese Academy of Sciences Beijing China; ^3^ National Key Laboratory of Earth System Numerical Modeling and Application University of Chinese Academy of Sciences Beijing China; ^4^ School of Mathematical Sciences University of Chinese Academy of Sciences (UCAS) Beijing China; ^5^ School of Engineering Science University of Chinese Academy of Sciences Beijing China; ^6^ College of Resources and Environment University of Chinese Academy of Sciences Beijing China

**Keywords:** alternative stable state, ecosystem hysteresis, model, positive‐negative feedback, regime shifts

## Abstract

Ecosystem hysteresis, the occurrence of catastrophic transitions due to external disturbances, is a prevalent phenomenon in dynamic ecosystems. Understanding hysteresis is essential for predicting ecosystem responses and developing effective restoration strategies. However, the intrinsic dynamics quantifying the positive‐negative feedback in driving hysteresis and its intensity remain undisclosed. We introduce a quantitative framework to address hysteresis by assessing ecosystem states and feedback loops, which diverges from prior phenomenological theories of hysteresis. Employing this framework, a generalized mechanism model is proposed to estimate positive‐negative feedback strengths and defines the irreversible potential of hysteresis to determine its intensity. We identify a dimensionless critical constant that indicates whether hysteresis occurs. The model effectively captures both forward and backward trajectories of hysteresis across various ecological scales. The direction of state transitions may be predicted using unidirectional data. Our findings offer a universal framework for predicting and mitigating catastrophic state shifts of ecosystems.

## Graphical abstract

A simplified model examines ecosystem transitions into alternative states after disturbances by leveraging positive and negative feedbacks. It identifies critical tipping points and quantifies hysteresis. Tests conducted on lakes, grasslands, and pitcher‐plant leaves align closely with empirical data and enable the prediction of recovery trajectories from unidirectional observational records.

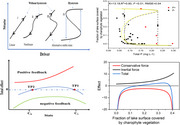

## 1 Introduction

Human activities and climate change increasingly risk inducing catastrophic transitions in ecological systems, commonly known as hysteresis [1, 2, 3, 4, 5, 6]. The occurrence of hysteresis leads to profound alterations to biodiversity, ecosystem functioning, and services [1, 7, 8]. Hysteresis is not limited to ecological systems, but widely observed in the diverse systems or processes, such as ferromagnetic magnetization [9], combustion process [10], human depression [11], and collapsed financial markets [12]. Fundamental understanding of hysteresis is crucial for accurately predicting and managing ecosystem responses to environmental changes [7, 8]. By exploring the intrinsic mechanisms governing transitions between stable states, identifying critical tipping points, and quantifying the intensity of hysteresis, we can develop the effective conservation strategies that promote stability and mitigate undesired state transitions.

**FIGURE 1:**
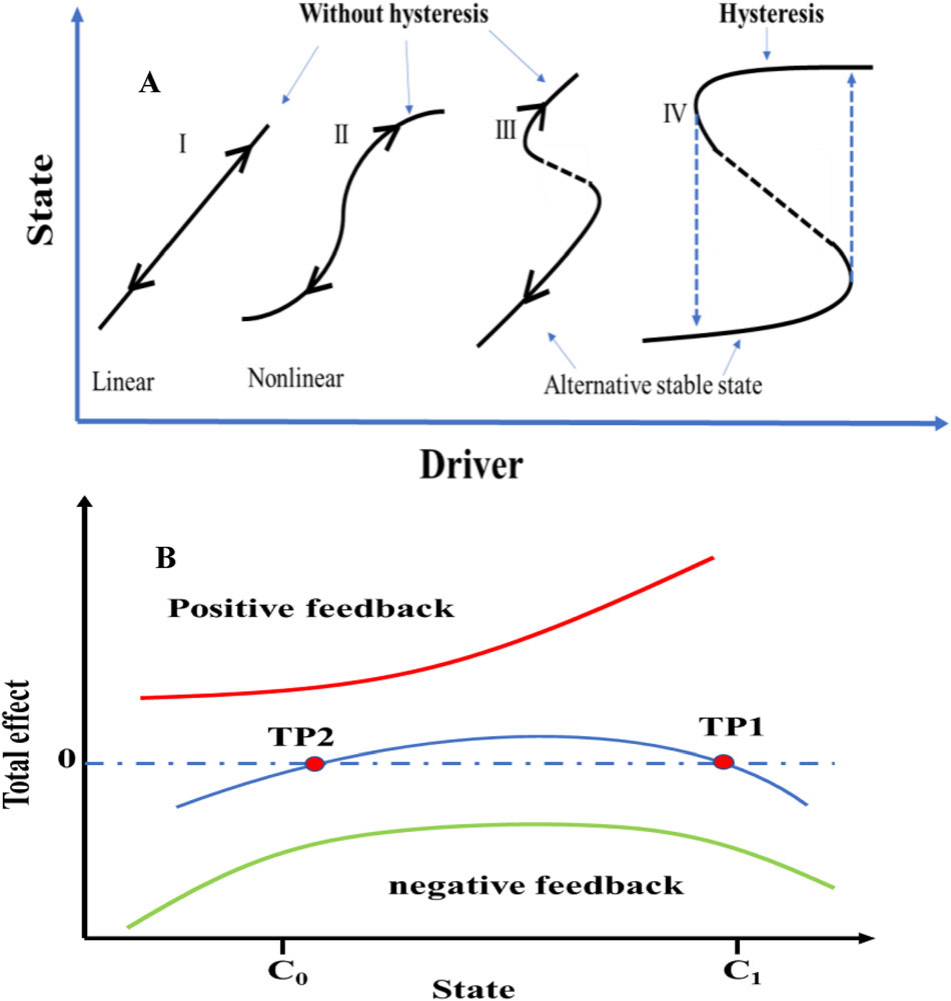
(A) Schematic of different types of state transitions with and without hysteresis in ecosystem. (I) The response of state in an ecosystem is linear to the changes in drivers and only one stable equilibrium exists; (II) As the drivers increase, the state continuously and gradually transit from an “upper” state to a “lower” state, which are mutually exclusive; (III) A sudden state transition between alternative stable states, there is no hysteresis occurs as the environmental drivers increase or decrease; (IV) A sudden catastrophic state transition between multiple stable states in a hysteretic ecosystem with distinct thresholds for forward and backward paths. (B) Schematic the mechanisms of positive and negative feedback for state transition in an ecosystem. Positive feedback pushes state forward, while negative feedback keeps stability. As environmental drivers favoring positive feedback increase, it exceeds negative feedback causing the system forward past a threshold (TP1) and shift to a new state. When the environmental stress is alleviated, the ecosystem moves backward from the new state, crossing another threshold (TP2) and returning to the initial state along an alternative path.

From the perspective of dynamic evolution, there is a significant commonality of hysteresis, which is often accompanied by ecosystem state transition: two competitive mechanisms with varying intensities simultaneously work on state transition. Shifts in the dominance of these mechanisms lead to diverse successional paths (Figure 1A). In addition, hysteresis is susceptible to different states due to its four intrinsic characteristics: boundedness, uniqueness, mutability, and irreversibility [1, 13, 14, 15, 16, 17]. Therefore, this susceptibility arises from a complex interplay of positive and negative feedback mechanisms [17, 18] (Table 1). For instance, inertial positive feedbacks can amplify minor perturbations, pushing the system past a tipping point into a new stable state [19, 20], while conservative negative feedbacks counteract changes to preserve the initial state. The quantification of these feedbacks is thus pivotal for the direct assessment of ecosystem hysteresis (Figure 1B). However, most studies have examined the hysteresis of the given ecosystems merely using phenomenological methods [15, 21]. They have described the observed behaviors, without explicitly simulating the underlying physical or mechanism processes or necessarily delving into the internal processes. A unified framework of intrinsic mechanism that governs hysteresis is still lacking [22, 23, 24, 25, 26]. Accordingly, there is a pressing need to predict hysteresis by establishing a mechanistic framework that links positive and negative feedbacks across diverse ecosystems.

**TABLE 1:** Glossary of terms.

Term	Definition	Refs.
Stable State	A relatively stable configuration of an ecosystem at a specific time, defined by its structure, function, and feedback mechanisms. It not only reflects the compositional characteristics of biotic and abiotic components but also includes the overall behavioral patterns of internal system processes.	[14, 27, 28, 29]
Regime shift	Large‐scale, abrupt, and persistent changes in the structure and function of an ecosystem, which are often difficult to reverse or even irreversible, typically triggered by external disturbances and dominated by positive and negative feedback.	[15, 30, 31]
Hysteresis	In ecological communities, hysteresis results in thresholds that, when crossed, cause abrupt shifts in biomass and biological diversity that cannot be readily reversed.	[32, 33]
Tipping Point	The critical point or area where an ecosystem suddenly transitions from one state to another. This transition is usually accompanied by fundamental changes in the structure and function of the ecosystem.	[34, 35]
Positive Feedback	The situation where changes in a certain part of an ecosystem cause a series of other changes, which in turn accelerate the changes in the part that initially changed. It often drives ecosystems away from equilibrium, which may lead to regime shifts or collapses and is the basis for the occurrence of hysteresis.	[19, 36]
Negative Feedback	The core mechanism for ecosystems to maintain stability and resist disturbances, which helps the system stay in its current state.	[37]

To address these issues, we propose a framework that can quantify the state transition of the ecosystem and the competition mechanism. Two stable states in a bistable system are initially modeled as distinct positive values, denoted *C*_0_ and *C*_1_, where 0 < *C*_0_ < *C*_1_. We denote the current state of the system as the variable *Y*, which satisfies the condition *C*_0_ ≤ *Y* ≤ *C*_1_. We use two functions of opposite monotonicity to characterize the two competing mechanisms, denoted as *h*(*Y*) and *g*(*Y*). The function ϕ(*g*,  *h*) is applied to characterize the interaction of the two mechanisms. Therefore, our framework is: $x=f\;\left(Y\right)=\phi \left(g(Y),h(Y)\right),Y\in \left[C_{0},C_{1}\right]$, where *x* is a driving factor. Next, *g*(*Y*), *h*(*Y*) and ϕ(*g*,  *h*) are concretized to achieve the mechanism model to capture the underlying mechanisms of state transitions of the hysteretic ecosystem. We emphasize that the proposed model is not intended to represent the detailed biological or ecological mechanisms—such as species interactions, dispersal limitation, or spatial heterogeneity—that underlie state transitions in real ecosystems. Rather, it is a feedback‐based mechanistic model that captures the net effects of positive and negative feedbacks on system state transitions. The model abstracts these feedbacks into two competing functions, allowing us to quantify the potential for hysteresis without explicitly simulating the underlying biological processes.

## 2 Generalized Mechanism Model in Brief

Based on the aforementioned perspective of system dynamics and the combustion theory (Figure S1), we concretized the framework as our model.

(1) $$x=f\;\left(Y\right)=\frac{Y-C_{0}}{C_{1}-Y}\;\times e^{\frac{K}{Y}}$$

where *K* is the ​irreversible potential that determines the performance of system state variables, playing a decisive role in measuring the divergence between the forward and backward trajectories of state transition (Figure S2). *e* is the Euler's number. A larger irreversible potential (*K*) signifies a stronger ecological resistance that needs to be overcome prior to the state transition.​ In our model, $g\;\left(Y\right)=\frac{Y-C_{0}}{C_{1}-Y}$ and $h\;\left(Y\right)=e^{\frac{K}{Y}}$ represent two competition mechanisms respectively, and ϕ (*g*, *h*) =  *g* · *h* characterize the interaction. A distinguishing characteristic of our model is the incorporation of the system state as an independent variable, in contrast to the driving factors. At first glance, the structure of this model may appear counterintuitive, however, it is crucial within the context of hysteresis‐driven ecosystems, where a single driving factor *x* is associated with two potential system states. The model demonstrates the intrinsic dynamics of systems by encapsulating the underlying mechanisms governing state transitions in systems with hysteresis and their interplay.

## 3 Sensitivity Analysis of the Model

**FIGURE 2:**
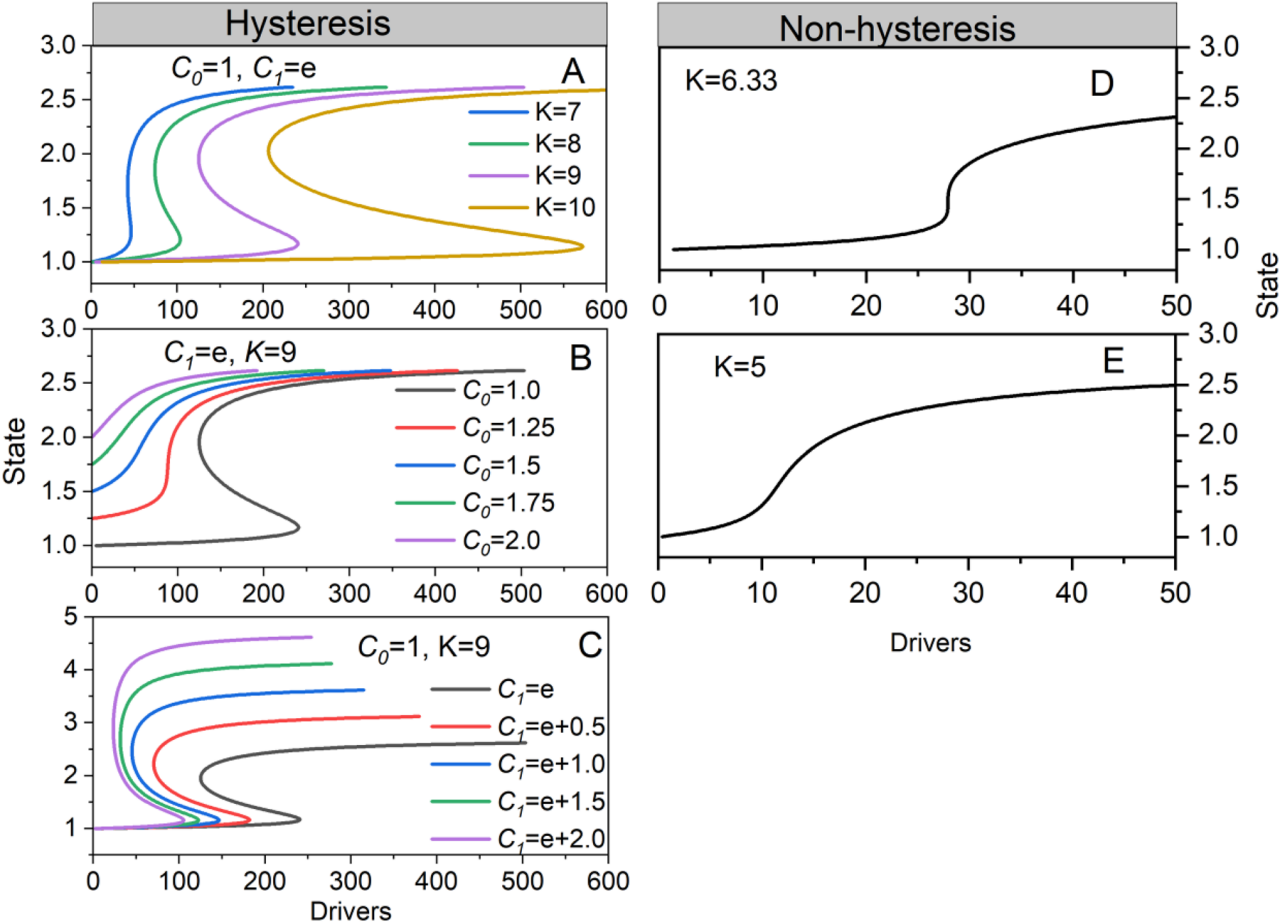
Sensitivity analysis of the uncertainty in alternative stable state with (A–C) or without (D–E) hysteresis as state shifts from an initial to another state and ​irreversible potential for the model. (A) Effect of irreversible potential *K* on the dynamics of state while parameter of an initial state (*C*_0_ =  1) and another state (*C*_1_ =  *e*) was fixed. (B–C) Effect of change in an initial and another state while ​irreversible potential is fixed to 9. When ​irreversible potential is less than or equal to critical constant (*K* ^*^ = 6.33), state transitions of ecosystem show three types of state stations without hysteresis phenomenon. (D) A sudden state transition between two mutually exclusive states; (E) a continuous and gradual state transition from the initial to another state as the drivers increase, such as environmental parameter.

To elucidate the influence of parameter variability on the evolution of hysteretic ecosystem states, we initially established the standardized form of our model (*C*_0_ =  1,  *C*_1_ =  *e*,  *K*  =  9) to conduct the sensitivity analysis of the model. The analysis showed that as *K* increases gradually, the intensity of hysteresis increases as well. This indicates that an increase in *K* results in a greater driving force being required for an ecosystem to undergo a state transition (Figure 2A). With the increase of the initial state, K^*^ increases, while the hysteresis is less likely to occur (Figure 2B), In contrast, with the increase of state *C*_1_, K* decreases, the hysteresis phenomenon is more likely to occur (Figure 2C). When *K* is less than or equal to the critical constant, there are two different types of state transitions in non‐hysteresis ecosystems (Figure 2D,E). As the driver increases, the state transitions continuously and gradually progress from the higher to the lower two states. We can conclude that the significance of our model lies not only in its applicability to ecosystems with hysteresis, but also in its relevance to ecosystems without hysteresis. Furthermore, beyond the realm of ecosystems, it can be effectively employed in other systems with hysteresis.

## 4 Results

**FIGURE 3:**
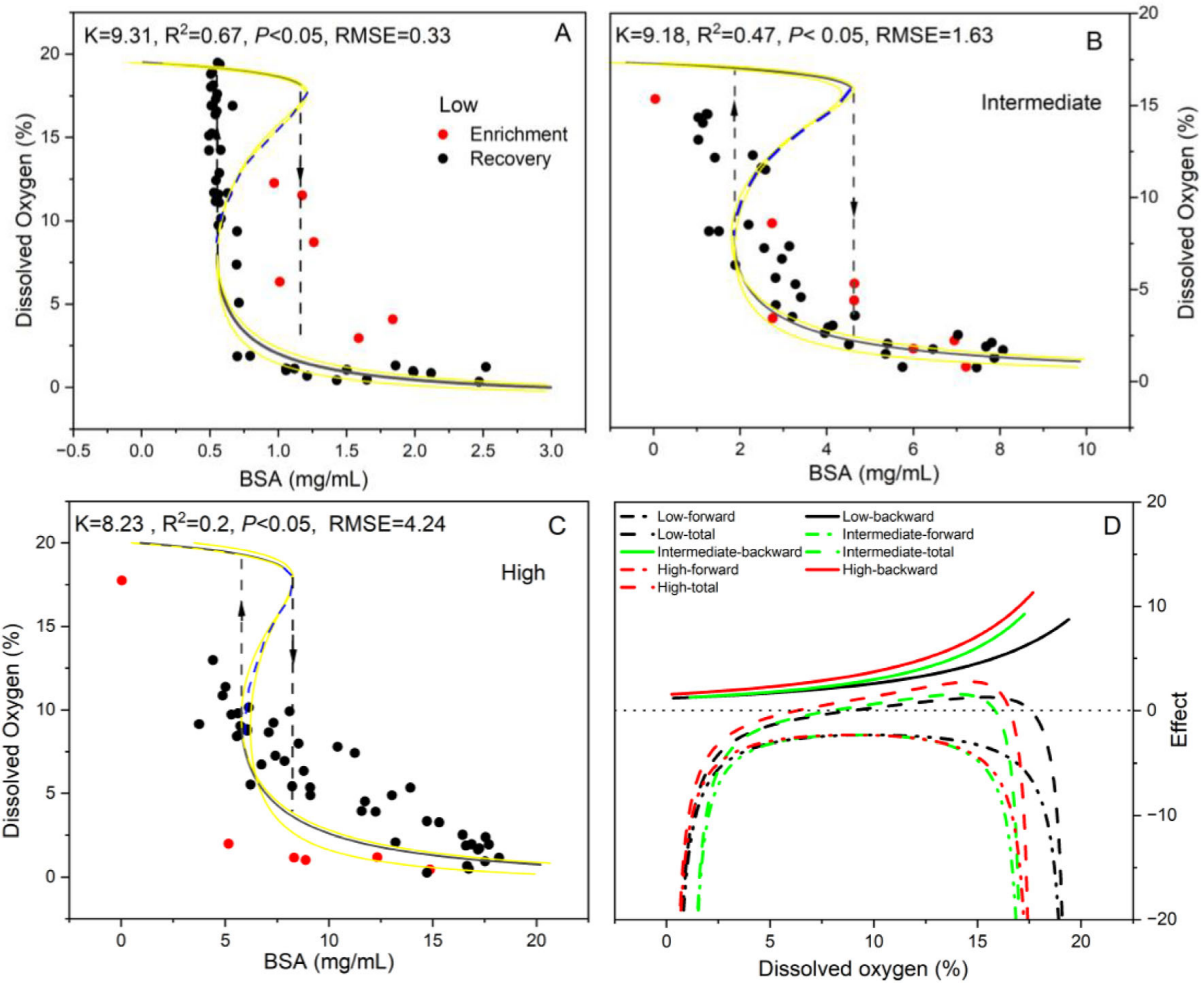
Counterclockwise and clockwise state hysteresis of dissolved oxygen by altering a single driver variable, bovine serum albumin (BSA) from low to high concentration in an aquatic ecosystem. (A–C). The fitted state hysteresis using our model. Down and up arrows indicate the observed and continuous change for state when BSA enrichment and recovery under three concentrations of BSA, respectively. Blue dash means the intermediate state of dissolved oxygen. (D–E) The relationship between the effect of inertial, conservative and total force caused by positive and negative feedback with state of dissolved oxygen under three concentrations of BSA [38]. The yellow solid line represents the 95% confidence interval in panels A–C. The boundaries of the confidence interval corresponding to the specific *K* value are provided at the following URL: https://github.com/WangXin21/Ecosystem‐hysteresis.

We first applied the model to an individual species and examined variations in dissolved oxygen (DO) of leaves of the pitcher plant *S. purpurea* under varying concentrations of undigested bovine serum albumin (BSA). As the BSA rates increased, the dynamics of DO range from low‐level clockwise hysteresis to moderate environmental tracking, and finally to high‐level counter‐clockwise hysteresis [38]. Application of our model revealed two distinct hysteresis patterns, each defined by unique ​irreversible potential values and specific tipping points. Specifically, treatment with a high concentration of BSA led to a more rapid transition in the DO state and a more expeditious recovery (Figure 3A–C). This finding suggests that a low ​irreversible potential is associated with a high BSA concentration. In line with this result, treatment with a low concentration of BSA resulted in the opposite effects (Figure S3; Table S2). The shifts in the stable states and the associated tipping points are driven by the interplay of positive and negative feedback. This interplay is evidenced by the intersections of the hysteresis patterns with the line representing zero net change (Figure 3D). Altogether, the model accurately predicts the observed shifts in dissolved oxygen concentrations in response to varying BSA concentrations.

**FIGURE 4:**
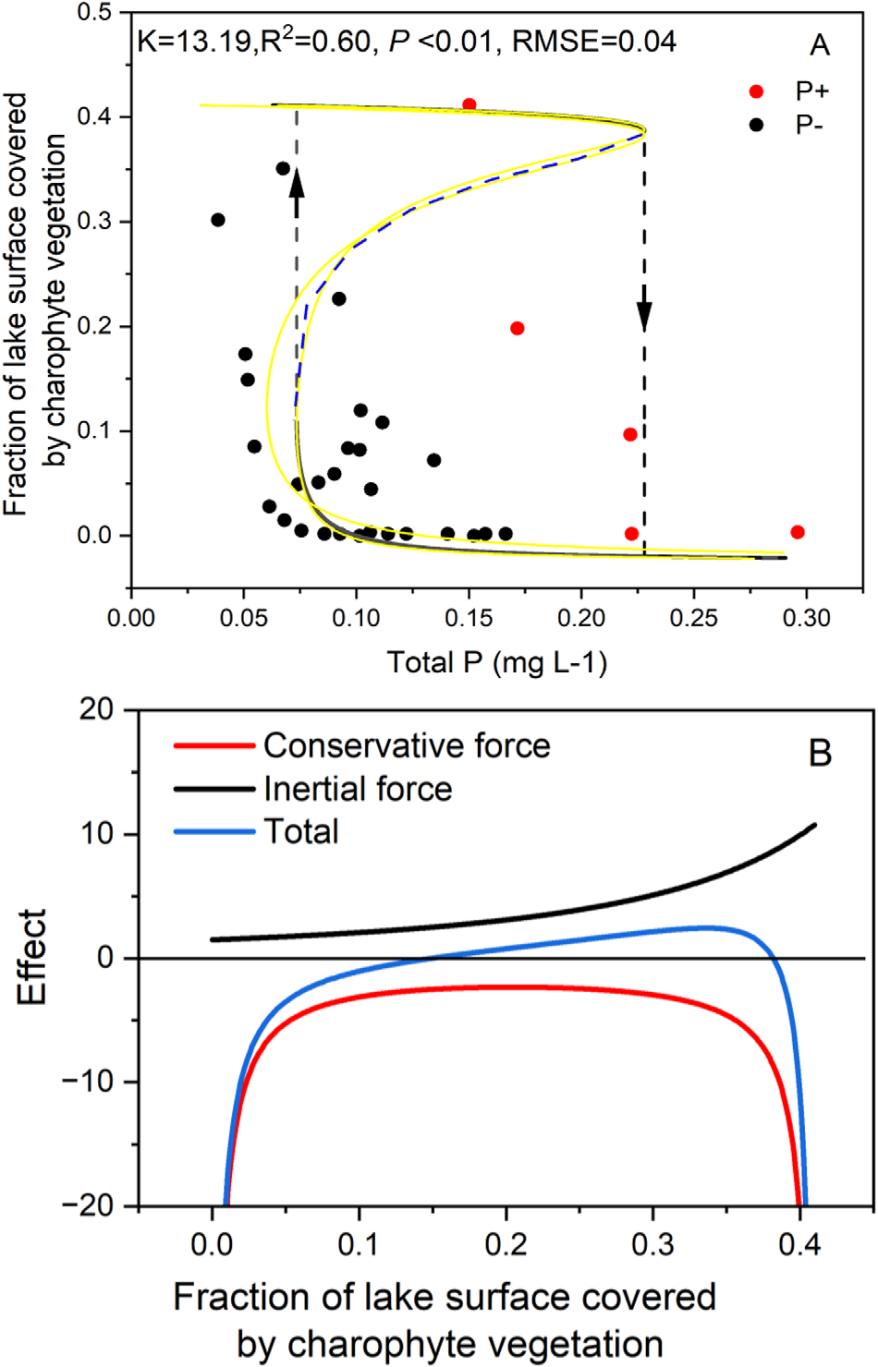
(A) The fitted hysteresis in the response of charophyte vegetation to increase (P+) and subsequent decrease (P‐) of the phosphorus concentration in the shallow Lake Veluwe. Down and up arrows indicate the abrupt and continuous change for state of charophyte vegetation when the phosphorus concentration increase and subsequent decrease, respectively. Blue dash means the intermediate state of charophyte vegetation. (B) The relationship between the effect of inertial, conservative and total force caused by positive and negative feedback with state of charophyte vegetation while the phosphorus concentration increases and decrease [39]. The yellow solid line represents the 95% confidence interval in panel A. The boundaries of the confidence interval corresponding to the specific *K* value are provided at the following URL: https://github.com/WangXin21/Ecosystem‐hysteresis.

The model for a lake ecosystem on a long‐term scale is presented as an example. The hysteresis was observed in response to the increase of charophyte vegetation in the shallow Lake Veluwe and the subsequent decrease of the phosphorus concentration [39]. The model parameter 𝐾 was estimated alongside other forces‐conservative and force‐inertial, that contribute to the system's dynamics. The results demonstrate that our model effectively captures the hysteresis dynamics of charophyte vegetation, as well as the forward and backward tipping points (Figure 4A). The substantial 𝐾 implies that it is difficult for the system to restore to its initial stable state through phosphorus concentration reduction alone. By applying our model, we disentangled the relative contribution of positive and negative feedback, including fish stock and other factors, to shifts in state within this lake ecosystem. Because the specific impacts of planktivorous fish predation on large zooplankton (or the fish stock's influence on hysteresis dynamics) remain indistinct (Figure 4B), our model provides insights into their combined effects on ecosystem stability. Furthermore, we also estimated the phosphorus concentration thresholds for the forward and backward tipping points to be 0.23 and 0.07 mg L^−1^, respectively.

**FIGURE 5:**
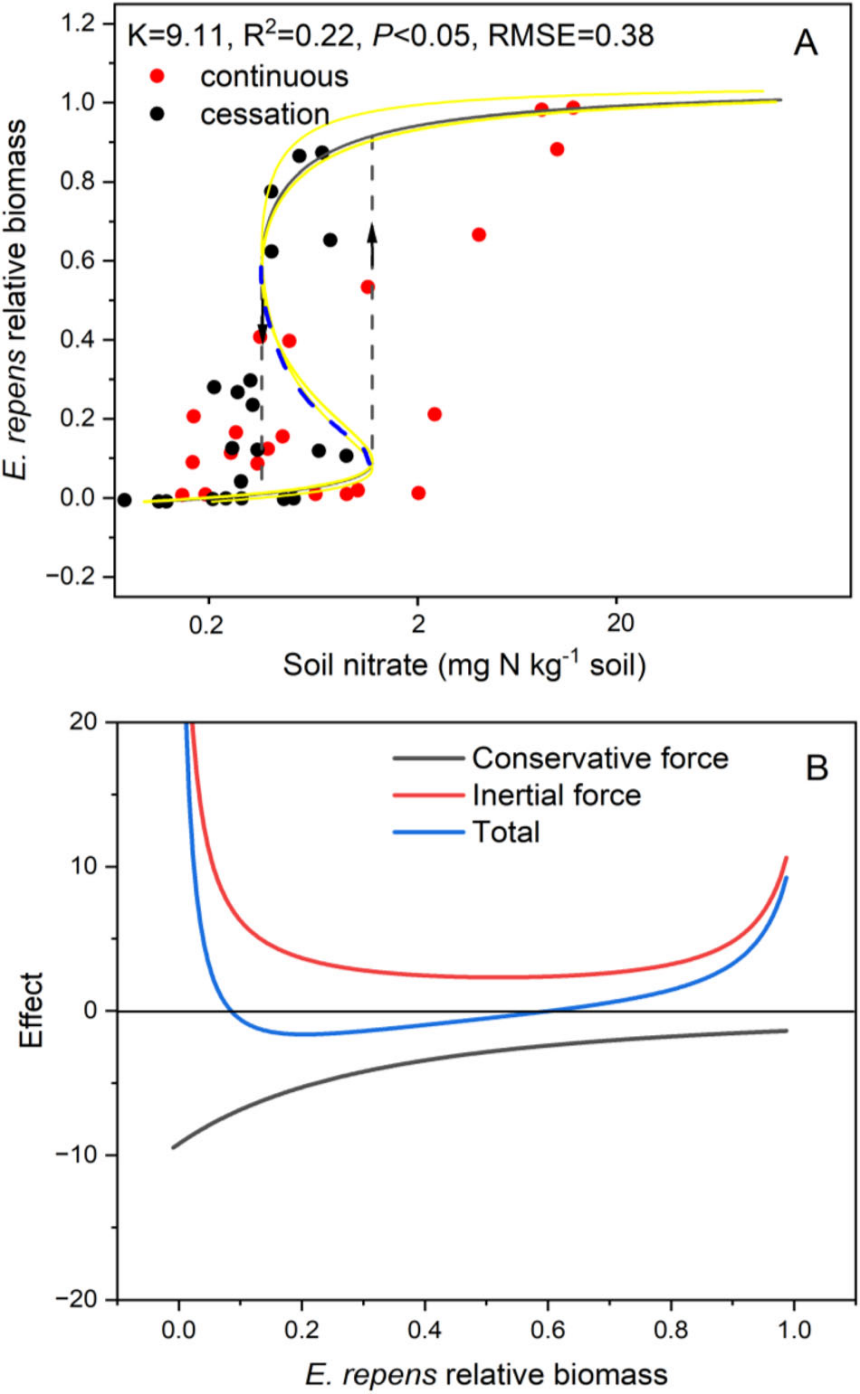
(A) The fitted discontinuous, hysteretic response of states of *Elymus repens* relative biomass to forward nitrogen addition and backward cessation of nitrogen addition in the terrestrial ecosystem. Up and down arrows indicate the abrupt and discontinuous change for state of *Elymus repens* relative biomass when the nutrient start consciously adding and cessate adding, respectively. Blue dash means the intermediate state of *Elymus* repens relative biomass. (B) The relationship between the effect of inertial, conservative and total force caused by positive and negative feedback with state of *Elymus repens* relative biomass when the nutrient starts consciously adding and cessate adding, respectively [3]. The yellow solid line represents the 95% confidence interval in panel A. The boundaries of the confidence interval corresponding to the specific *K* value are provided at the following URL: https://github.com/WangXin21/Ecosystem‐hysteresis.

To exemplify our model for a terrestrial ecosystem operating on an extended time scale, we conducted an analysis of a plant diversity dataset from a 30‐year grassland experiment, which encompassed a 10‐year nitrogen addition phase followed by a 20‐year cessation period [3]. The model simulation indicated that while the relative biomass of *E. repens* exhibited both forward and backward tipping points, the significant *K*  =  9.11 suggests that plant diversity cannot be restored to its pre‐enrichment levels merely by ceasing high rates of nitrogen input alone; it will require an extended period or additional restoration measures (Figure 5). The fitted model, therefore, underscores the complexity of ecological recovery following prolonged disturbance and highlights the importance of considering catastrophic processes in the management and conservation of ecosystems.

**FIGURE 6:**
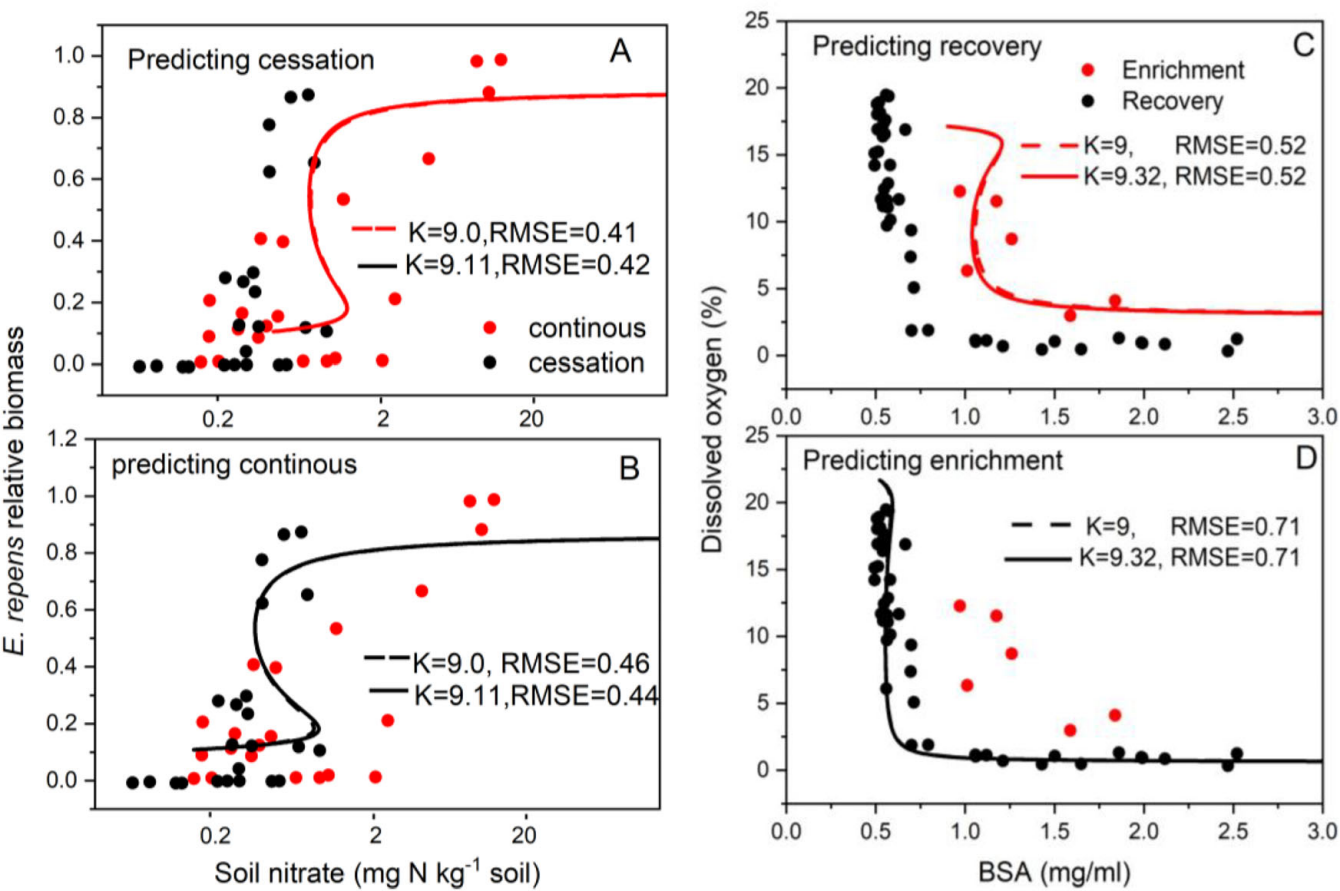
The prediction of the backward or forward path of state of ecosystem with hysteresis base d on only forward or backward measured data. (A,B) and (C,D) The data is from Figure 3A and Figure 4.

Solely based on the dataset of forward switching, the prediction of the trajectory and critical points of backward switching presents a significant challenge. In order to apply our model for predicting potential recovery trajectories and critical points, we conducted simulations on two distinct datasets: one for plant diversity shifts as mentioned above, and the other for low‐level dissolved oxygen shifts in individual species (Figure 6). Our model effectively predicted the dynamics of backward transition, along with the critical points, by utilizing only the unidirectional dataset of forward transition.

## 5 Discussion

In recent decades, researchers have made significant advancements in understanding the underlying mechanisms for ecosystem hysteresis [33, 40]. One of the key factors is the interplay of positive and negative feedback loops, which suggests that a system's final state is influenced by its historical conditions, and that there exist dual response mechanisms to driving parameters [17, 41]. The initial response mechanism for forward‐driving variables may be either amplified or diminished in the reverse mechanism for backward‐driving variables, potentially leading to the emergence of two equilibrium states that bridge the initial and final (another) state parameters. Divergent successional pathways may arise from the varying intensity of these forward and backward mechanisms, as well as from the system's intrinsic properties. If the key parameters of the final stable state show minimal deviation from those of the initial state, or if the system's response is markedly pronounced, the evolution may proceed smoothly with a single stable state. In contrast, a weak system response can lead to a more turbulent and abrupt successional process, characterized by multiple stable states [38, 42]. Such drastic successional changes in key parameters can trigger diverse system responses to driving variables, resulting in both forward and backward shifts and potentially leading to multi‐branching outcomes. The classical models, such as Threshold Generalised Additive Models (TGAMs), Global Climate Models (GCM), and Zeeman's cusp catastrophe model, lack a universal metric for quantifying hysteresis intensity, they cannot be directly estimated from field data, and they do not support cross‐system comparisons [43, 44, 45]. Unlike these models, our mechanism‐driven model excels in parameter acquisition and mechanistic clarity. It quantifies hysteresis intensity via multiplicative positive/negative feedback interactions. The dimensionless parameter *K* enables direct cross‐system comparison and measures hysteresis strength (loop width), while threshold K* clearly distinguishes hysteresis from non‐hysteresis regimes. Irreversible potential *K* further severs as a diagnostic of restoration difficulty: the larger *K* becomes, the longer the system must battle internal feedback resistance and the harder recovery proves to be, thereby supplying a mechanistic benchmark for timing ecological restoration.

The primary strength of our model lies in its ability to quantitatively capture the interplay between positive and negative feedback forces that drive ecosystem hysteresis. This dynamic stability can be visualized as a “tug‐of‐war” game between two opposing forces (Figure 1B). Specifically, a state transition induced by a disturbance is characterized by a transient dominance of positive feedback that overwhelms the asymmetrically weakened negative feedback, thereby pushing the system past the forward tipping point (TP_1_) [17]. The return transition is impeded because the negative‐feedback barrier re‐strengthens along the alternate trajectory, an asymmetry that our model captures through the irreversible‐potential parameter *K* (see Figure 1B and Equation 2). Our study advances the understanding of these combined effects by showing that the relative magnitudes of positive and negative impacts from disturbances are governed by a universal function. This function is distinguished by two phases: an initial forward phase that establishes a new stable state, and a subsequent backward phase that may recover the system to its initial state. We observed that the shape of this function demonstrates universality across multiple systems, while its parameters provide informative insights into the dynamic hysteresis phenomenon. These findings quantitatively reinforce the theoretical constructs of positive and negative feedback, thus deepening our understanding of the mechanisms governing hysteresis production. Our model's innovation lies in its divergence from traditional differential equation models for hysteresis, such as the Hill‐function and Polynomial function, which are often data‐intensive but lack universal applicability [21].

To assess the strength of hysteresis, researchers have long‐term employed stability or resilience as metrics [33]. However, the essence of hysteresis is the deviation of the return path from the initial trajectory when transitioning between system states. Therefore, the degree of this deviation can be considered as a robust indicator for detecting the presence and intensity of hysteresis [33], diverging from traditional resilience assessments. In our study, we propose a universal critical constant (K^*^ = 6.33) to identify the occurrence of system hysteresis. Our results also suggest that an increase in *K* leads to a corresponding enhancement in hysteresis intensity and demands extra energy for recovery in turn, thereby making it more challenging for the system to restore to its initial state (Figures S3 and S4; Table S2). In ecosystem hysteresis, the presence of ​irreversible potential implies that ecosystems may not be able to fully recover to their initial state; instead, they may undergo prolonged recovery period [38]. This complexity arises because the structure, function, and composition of ecosystem are shaped by myriad factors, including species interactions, environmental conditions, and human activities. Severe ecosystem damage can lead to changes in these factors, potentially preventing a return to the initial state. The ​irreversible potential, therefore, provides an intuitive framework for comparing different systems and for pinpointing the key factors that hinder recovery.

Scientists are working on identifying the tipping points (TPs) of ecosystems beyond which hysteresis can occur [46, 47, 48]. The prediction of forward and backward shifts' TPs remains a challenge based on the available system data. Based on the forward shift dataset of the system, our model addresses this challenge not only by identifying two distinct TPs, but also by enabling the prediction of the backward TPs. This capability is particularly valuable for ecological forecasting and management. Nonetheless, we emphasize that the tipping points identified here are mathematical bifurcation points arising from the abstracted feedback functions *g*(*Y*) and *h*(*Y*). They should be viewed as diagnostic indicators of potential regime shifts, not as predictive statements about the exact ecological processes (e.g. species re‐assembly, seed‐bank dynamics, spatial rescue, or nutrient legacy) that will cause the shift in any specific ecosystem. Empirical application therefore requires (1) careful selection of state and driver variables that are demonstrably linked to the dominant feedbacks of the system, and (2) complementary field or experimental data to account for additional ecological mechanisms, such as time‐lags, spatial heterogeneity, or alternative limiting factors [44, 49, 50]. The primary limitation of the current model is its inability to explicitly account for the temporal dimension, which limits its capacity to quantitatively determine the timing of state transitions. While the model can qualitatively assess recovery difficulty through the irreversible potential 𝐾, it lacks the means to specify when such transitions occur. Future work will therefore focus on incorporating the time dimension by introducing the rate of change of driving forces into the framework.

## 6 Methods

### 6.1 Example Data Sets

#### 6.1.1 Dissolved Oxygen (DO) of Leaves of S. Purpurea

This dataset derived from enriched pitcher‐plant leaves of *S. purpurea* in a replicated greenhouse experiment. The experiment was conducted in 2015 and 2016 at the University of Vermont's Biological Research Complex in a temperature‐controlled greenhouse. In our study, we analyzed the data of Figure 3 [38]. By altering a single driver variable, bovine serum albumin (BSA), elicits counterclockwise and clockwise hysteresis.

#### 6.1.2 Charophyte Vegetation Hysteresis in a Lake Ecosystem

This dataset comes from a classic state transition study in a shallow Lake Veluwe. Hysteresis in the response of charophyte vegetation was found as the increase and the subsequent decrease of the phosphorus concentration. The data were recorded in the late 1960s and early 1970s [39].

#### 6.1.3 Biodiversity State of a Grassland Ecosystem

The dataset consists of the variations in plant diversity of a grassland ecosystem after the cessation of the nitrogen addition [3]. This dataset spans a period of 30 years (from 1982 to 2011) in a long‐term nitrogen addition and cessation study in a successional grassland at Cedar Creek Ecosystem Science Reserve. Plant species number and biomass in each plot were measured every year from 1982 to 1994, at least two of every three years from 1995 to 2004, and in 2008 and 2011. At the same time, nitrate concentration in soil cores was also measured during 1985, 1989, 1994, and 2002.

### 6.2 Quantify the Two Competitive Mechanisms

These two mechanisms are well described by positive and negative feedback in system theory. The analysis for the state *C*_0_ and *C*_1_ is similar, without loss of generality, assuming that the system is at *C*_0_ and moving toward *C*_1_. The positive feedback drives the system to *C*_1_ is formed as $\frac{dY}{dx}>0$, which corresponds to the monotonically increasing property of the function *x*  =  *g*(*Y*),  or *Y*  = *g* ^−1^ (*x*). This indicates that the driving factor moves in the same direction as the system state does. Negative feedback is modeled as *x*  =  *h*(*Y*) *or* *Y*  = *h* ^−1^ (*x*), which is monotonously decreasing, suggesting that the system state moves in the opposite direction of the driving factor change. The ultimate direction of the system state is determined by the derivative of the interaction $\frac{d\phi (g(Y),\;h(Y))}{dY}$. Therefore, tipping points correspond to the points where their derivative is equal to 0. In the following function, we let $g\;\left(Y\right)=\frac{Y-C_{0}}{C_{1}-Y}$ and $h\;\left(Y\right)=e^{\frac{K}{Y}}$, ϕ (*g*,  *h*) =  *g* · *h* to obtain the hysteresis model:

$$x=f\left(Y\right)=\frac{Y-C_{0}}{C_{1}-Y}\;\times e^{\frac{K}{Y}}$$

As shown in the above analysis, our model can govern not only the system with hysteresis, but also the system without hysteresis. To find the tipping points of ecosystem transition, it is necessary to take the derivative of the *f*(*Y*) and set *f*′ (*Y*) =  0. The presence of hysteresis thus equates with the existence of two distinct real roots in the quadratic equation, as shown below:

(2) $$K>\frac{4C_{1}C_{0}}{C_{1}-C_{0}}$$

When *K* ≤ K*, ($K^{*}=\frac{4C_{0}C_{1}}{C_{1-}C_{0}}\;$ K* is critical constant, which is a dimensionless number, to characterize the critical point that determines hysteresis or not (See *Supplementary* for further details).

### 6.3 Application of the Model

First, our model is normalized to the following form (see *Supplementary* for why and how to normalized the model).

$$x=f\left(Y\right)=\frac{Y-1}{e-Y}\;\cdot e^{\frac{K}{Y}}$$

Then, we define the one‐sided state transition function based on the model. Taking the state transition from state 1 to state *e* as an example, for a fixed *K*, we calculate its TP point $\left(x_{TP_{1\to e}}^{m_{K}},\;y_{TP_{1\to e}}^{m_{K}}\right)$, and then make an *x*‐axis vertical line over it. The vertical line intersects with the model curve, and we note the intersection point as $\left(x_{TP_{1\to e}}^{m_{K}},\;y_{x_{TP_{1\to e}}^{m_{K}}}^{m_{K}\;}\right)$. We use the following function as a model for state transfer.

$$x=\alpha_{1\to e,K}\;\left(Y\right)=\left\{\begin{array}{cc} \frac{Y-1}{e-Y}\cdot e^{\frac{K}{Y}}, & Y\in \left(0,y_{TP_{1\to e}}^{m_{K}}\right]\\ x_{TP_{1\to e}\;}^{m_{K}}, & \;Y\in \left(y_{TP_{1\to e}}^{m_{K}},y_{x_{TP_{1\to e}}^{m}}^{m_{K}\;}\right]\\ \frac{Y-1}{e-Y}\cdot e^{\frac{K}{Y}}, & \;Y\in \left(y_{x_{TP_{1\to e}}^{m_{K}}}^{m_{K}\;},e\right) \end{array}\right.$$

Similarly, the transfer function on the other side is denoted as $\alpha_{e\to 1,K}\left(Y\right)$.

### 6.4 Estimating ​Irreversible Potential Based on Measured Data

We employ the empirical risk minimization to estimate parameter *K*. Specifically, considering a system where one complete hysteresis loop has been recorded, we denote the experimental dataset as $\left\{\left(x_{i,1\to e},\;y_{i,1\to e}\right),\left(x_{i,e\to 1},\;y_{i,e\to 1}\right)\;\right\}$. Then we perform a standard transformation φ on $\left\{y_{i,1\to e},y_{i,e\to 1}\right\}$ and linear transformation ϕ_*K*_ on $\left\{x_{i,1\to e},x_{i,e\to 1}\right\}$ (See *Supplementary* for φ and ϕ_*K*_ specifications) and then compute the loss $l\;\left(K\right)=\sum_{i}{\left(\alpha_{1\to e,K}\left(\varphi (y_{i,1\to e})\right)-\phi_{K}\left(x_{i,1\to e}\right)\right)}^{2}+\sum_{i}{\left(\alpha_{e\to 1,K}\left(\varphi (y_{i,e\to 1})\right)-\phi_{K}\left(x_{i,e\to 1}\right)\right)}^{2}$. *K* of this hysteresis system is $\arg\min_{K}l\left(K\right)$. Let *n* denote the number of data points, and RMSE is calculated by $RMSE=\sqrt{\frac{l(K)}{n}}$. Once 𝐾 is determined, an inverse transformation is applied to convert the model points back to obtain a curve representing the original data.

### 6.5 Prediction Using the Model

Assuming we only have data points for a unidirectional forward or backward state transfer. Without loss of generality, we consider a transfer from state 1 to *e* and denote it as ${\left\{\left(x_{i,1\to e},y_{i,1\to e}\right)\right\}}_{i=1,\text{…}}$, and tipping point is $\left(x_{TP_{1\to e}},\;y_{TP_{1\to e}}\right)$. A value to *K* in advance is assigned for our model. We then obtain the data points of model ${\left\{\left(x_{i}^{m},\;y_{i}^{m}\right)\right\}}_{i=1,\text{…}}$. Next, we perform an inverse standard transformation φ^−1^ on $\left\{y_{i}^{m}\right\}$. Regarding alignment on the *x*‐axis, the *x*‐axis data is linearly transformed by utilizing the maximum and minimum values and the tipping point, followed by averaging the results.

$$\phi_{1}^{-1}\;\left(x\right)=\frac{\max_{i}\left(x_{i}^{1\to e}\right)-x_{TP}^{1\to e}}{\max_{i}\left(x_{i}^{m}\right)-x_{TP_{1\to e}}^{m}}\;\left(x-x_{TP_{1\to e}}^{m}\right)+x_{TP}^{1\to e}$$

$$\phi_{2}^{-1}\;\left(x\right)=\frac{\min_{i}\left(x_{i}^{1\to e}\right)-x_{TP}^{1\to e}}{\min_{i}\left(x_{i}^{m}\right)-x_{TP_{1\to e}}^{m}}\;\left(x-x_{TP_{1\to e}}^{m}\right)+x_{TP}^{1\to e}$$

$$trans_{x}\;\left(x\right)=\frac{\phi_{1}^{-1}\left(x\right)+\phi_{2}^{-1}\left(x\right)}{2}$$

The tipping point for the other side is $\left(trans_{x}\left(x_{TP_{e\to 1}}^{m}\right),\varphi^{-1}\left(y_{TP_{e\to 1}}^{m}\right)\right)$.

For systems that have not yet exhibited signs of transition, the prediction of tipping points relies on the estimation of *K*. A key advantage of our model is the cross‐system comparability of *K*, which enables consistent comparison across diverse systems. The application of the irreversible potential *K* must be strictly delimited: it is applicable exclusively to systems exhibiting analogous feedback mechanisms, and its threshold significance is subject to the historical interference and environmental regulation of specific ecosystems [51]. If a well‐studied lake with a known irreversible potential (*K*_1_) shares critical characteristics with a system of interest, *K*_1_ can serve as a prior estimate to inform proactive management strategies. Furthermore, ecosystems with similar critical characteristics may define a range of *K* values, offering upper and lower bounds to support future predictions for analogous systems. This normalized modeling framework offers a novel contribution to scalable ecological forecasting by enabling systematic and generalizable predictive capabilities.

## Author Contributions

Y.H., Y.F.W., and J.L. conceived and designed the projects; X.W. and Y.H. analysed the data. Y.H. and X.W. wrote the first draft of this manuscript; other authors provided editorial advice.

## Conflicts of Interest

The authors declare no conflicts of interest.

## Supporting information




**Supporting File**: advs73800‐sup‐0001‐SuppMat.docx.

## Data Availability

Data available within the article or its supplementary materials
