# Supplementary material for: Generalized Mechanism Model for Ecosystem Hysteresis
Source: Adv Sci (Weinh). 2026 Jan 14;13(17):e09008. doi: 10.1002/advs.202509008 (PMC13042370; doi:10.1002/advs.202509008)
Supplement: Supplementary file 1 — Supporting File: advs73800‐sup‐0001‐SuppMat.docx. [file ADVS-13-e09008-s001.docx]

Supporting Information for

**Generalized mechanism model for ecosystem hysteresis**

Yanbin Hao^1,2,3†^, Xin Wang^4†^, Jie Liu^5†^, Mingzi Wu^2^, Jianqing Du^1,3^, Kai Xue^6^, Xiaoning Song^6^, Xiaoyong Cui^2^, Tong Zhao^3,4^, Yanfen Wang^2,6^*

Corresponding author: [yfwang@ucas.ac.cn](mailto:yfwang@ucas.ac.cn)

**Development of generalized mechanism model**

Regime shifts in ecosystems are often characterized by an abrupt transition from one stable state to another once a driver crosses a critical threshold. Ecosystems exhibit self-organized criticality, meaning that even minor disturbances can induce abrupt and significant transformations in their structure and function. This can lead to destabilization. As dissipative structures, ecosystems depend on a continuous flow of energy and matter from their surroundings. The unique structure and function of these systems are shaped by intricate interactions between biological and non-biological processes, which undergo a continuous exchange of matter and energy. The ecosystems are thus intricate entities where diverse processes work together to maintain their internal thermodynamic cycles. A classical example is the shift between a clear-water, charophyte-dominated state and a turbid, phytoplankton-dominated state in shallow lakes (Scheffer et al. 2001). When phosphorus (P) is gradually increased and then decreased, the forward and backward shifts occur at different thresholds, producing the characteristic hysteretic loop (Fig. S1B). Similarly, in the field of combustion science, the theory of hot combustion characterizes ignition as a sudden and significant acceleration in the rate of chemical reactions that can occur either spatially or temporally. The analogy can be drawn between this concept and the hysteresis observed in ecosystems, where abrupt changes can trigger either ’catch fire’ or ‘shut down’. The mathematics that governs both situations is identical. Below we derive the generic curve once using the combustion notation (Box S1and Table S1) and once using the ecological notation (main text); the reader only interested in ecology can skip Box S1 without loss of continuity.

Box S1: A typical combustion derivation is as follows:

$Mc_{p}\frac{dT}{dt}=\dot{m}c_{p}\left( T_{u}-T_{0} \right)+Vq_{F}\dot{\omega}_{F}$ (1)

$M\frac{dY_{F}}{dt}=\dot{m}\left( Y_{F,u}-Y_{F,0} \right)-V\dot{\omega}_{F}$ (2)

$\dot{\omega_{F}}=\rho Y_{F}Ae^{\frac{{-E}_{a}}{R^{0}T}}$ (3)

$D_{a}=\frac{\frac{M}{\dot{m}}}{\frac{1}{A}}$ (4)

where *V* is the reactor volume. $\dot{m}$ is the mass flow rate. $c_{p}$ is the specific thermal capacity at constant pressure $T_{0}$ and $T_{u}$ are the temperature of the initial mixture and after reaction. $Y_{F,0}$ and $Y_{F,u}$ the mass fraction of the reactant in the initial mixture and after reaction. $\dot{\omega}_{F}$ is the component mass change rate. $E_{a}$ is activation energy, $\rho$ is the component density*.* $D_{a}$ is the collision Damköhler number.

Then, we standardize the equation (1) and (2) and get nondimensional form as:

$\frac{dT}{dt}=1-T+q_{F}D_{a}Ye^{\frac{{-T}_{a}}{T}}$ (5)

$\frac{dY}{dt}=1-Y+D_{a}Ye^{\frac{{-T}_{a}}{T}}$ (6)

For Da = 0, the chemically frozen flow limit is showed. By increasing Da along the lower branch representing the slow reaction rates, we cover all possible weakly reactive, nearly frozen, states the system can have. However, beyond $Da_{I}$ the system abruptly jumps to the upper branch existing for higher Damköhler numbers and faster reaction rates. Hence, point I is identified as the state of ignition and $Da_{I}$ is the ignition Damköhler number. If we next move along the upper branch with decreasing Da, then the system again jumps at point E back to the nearly frozen, lower branch. Therefor point E represents as the extinction state and $Da_{E}$ is the corresponding extinction Damköhler number. There exist three possible solutions for $Da_{E}<Da<Da_{I}$. Within this regime, the solution on the middle branch is unstable and regarded to be physically unrealistic. The distinction between the relevant solutions on the lower and upper branches can only be made by knowing the initial conditions.

Distinct ignition and extinction states can be illustrated by plotting either the reaction temperature or the burning rate against the system's Damköhler number (Da), as depicted in Fig. S1A.

If the system reaches the stability ($\frac{dT}{dt}=0)$, and then we get the solution of stable steady of Equation (5) and (6):

$D_{a}=\left[ \frac{(T-1)}{(1+q_{F}-T)} \right]\times e^{\frac{T_{a}}{T}}$ (7)


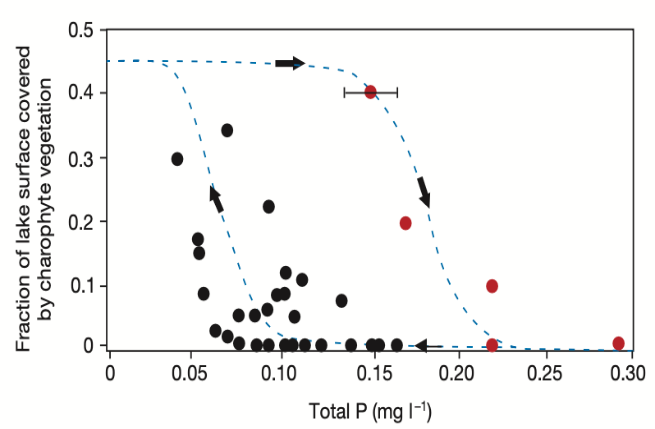


B


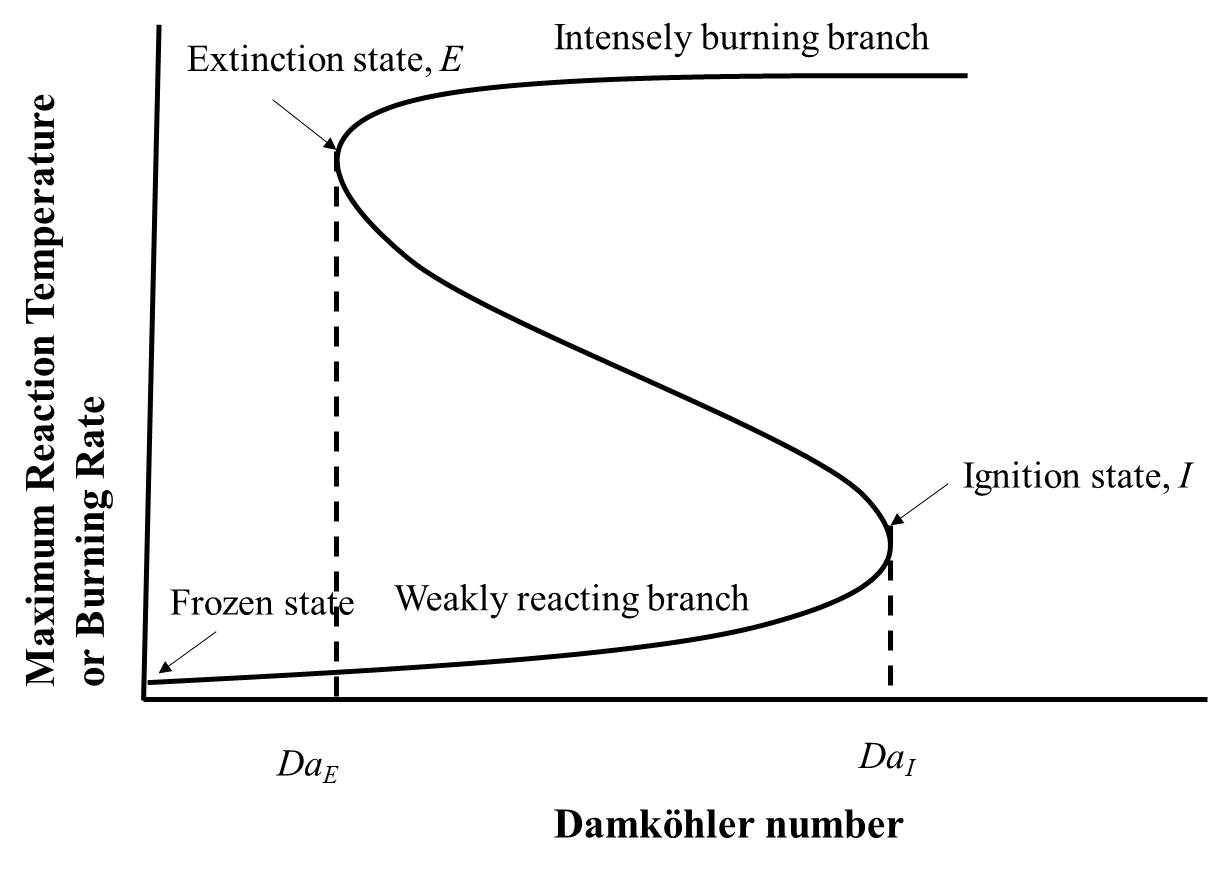


A

Figure S1. A. Classical combustion S-curve (temperature vs. Damköhler number) showing ignition (I) and extinction (E) points. B. Ecological analogue: fractional cover of charophytes versus total-P exhibiting the same folded geometry with forward and backward tipping points.

TableS1: One-to-one mapping between combustion and ecological terms used in the derivation.

| Combustion variable | Ecological | Interpretation |
| --- | --- | --- |
| Reaction temperature $T$ | Ecosystem state $Y$ | Current value of the state variable |
| Damköhler number $Da$ | External driver $x$ | Driver control variable |
| Ignition point $I$ | Forward tipping point | Threshold where the system jumps to the alternative state |
| Extinction point $E$ | Backward tipping point | Threshold where the system recovers to the original state |

**Framework of the model**

Building on the universality of both, we write the stable-state locus of the ecosystem driver $x$ as a function of the state variable $Y$:

$$x=\frac{Y-C_{0}}{C_{1}-Y}\cdot e^{\frac{K}{Y}}$$

Firstly, the two stable states in a bistable system are initially modeled as distinct positive values, denoted $C_{0}$ and $C_{1}$ where $0<C_{0}<C_{1}$. The current state of the system is denoted as the variable $Y$, satisfying the condition $C_{0}\leq Y\leq C_{1}$. When $Y<C_{0}$ or $Y>C_{1}$, the attraction to *Y* each of the two stable states is codirectional, leading to a rapid transition of the ecosystem towards either $C_{0}$ or $C_{1}$, depending on their proximity to *Y*. However, when $C_{0}\leq Y\leq C_{1},$ the system's potential is bidirectional, indicating the possibility for the ecosystem to either transition towards an alternative stable state or return to its initial state. Our proposed model integrates a monotonically increasing function with a monotonically decreasing one, facilitating an accurate estimation of the system's inclination towards either stable state, $C_{0}$ or $C_{1}$*,* based on the current state $Y$*.*

Secondly, we assume that the system is currently in the steady state $C_{0}$. The positive feedback should exhibit a monotonically increasing relationship with respect to $Y$, denoted $g(Y)$. Similarly, the negative feedback should be a monotonically decreasing function, denoted $h(Y)$. The coexistence of two feedbacks always entails intricate interactions, necessitating the employment of multiplication by combining $g(Y)$ with $h(Y)$.

Therefore, our model framework is：

$$x=g\left( Y \right)\cdot h\left( Y \right)，Y\in[C_{0},C_{1}]$$

where $x$ is driving factors, such as temperature, rainfall or nutrition contents. $Y$ is the current state of system. $C_{0}$ and $C_{1}$ are two stable states of system. $g\left( Y \right)$ and $h\left( Y \right)$ are the feedback functions. It is worth noting that mathematically we have chosen $g\left( \cdot\right)$ and $h\left( \cdot\right)$ as two functions with different rates of change (e.g., exponential increase and fractional linear decrease), this is to avoid the trivial cases where the multiplication is constant or always monotonic ($g\left( Y \right)=Y^{3},h\left( Y \right)=Y^{-3}$).

We introduce $g(Y)=\frac{Y-C_{0}}{C_{1}-Y}$ as the positive feedback relative to the system state $Y$ , which monotonically increase. Similarly, we define $h(Y)=e^{\frac{k}{Y}}$ as the negative feedback, which monotonically decrease. The transition of the system state is conceptualized as the outcome of a competition between these two feedbacks. We employ the product of these two functions with opposing monotonic behaviors to represent this competitive dynamic, encapsulating the essence of state shifts within our model.

**Irreversible potential and required energy for hysteresis**

In ecosystems exhibiting hysteresis, a key feature is the presence of two distinct tipping points, marking transitions between stable states. The remarkable fact is that both states are perfectly stable ($C_{0}$ and $C_{1}$). There are no spontaneous transitions from either state towards the other. The reason is that two stable states are not neighboring states. The transition from one to another can only take place over intermediate states which have greater driving forces (positive feedback or energy) than either of them. To put it crudely, one state must to be pushed away from its original position ($C_{0}$) to another ($C_{1}$) due to the given disturbance or energy supply. They are called the minimum disturbance force or activation energy- the force or energy needed to “push” or “activate” the transition. Call A* the disturbance force or activation energy, the excess force or energy needed in a transition in order that the transition may really occur (Fig. S2). This transition requires the system to surpass a high energy threshold, without which the state change cannot occur. Thus, the energy threshold (denoted as *A**) required for state transitions represents the energy input needed to propel the system from its initial state to a critical threshold (see the blue arrows in Fig. S2). There is a distinct possibility that the state $C_{0}$ and $C_{1}$ will undergo separation. In order to get this, it not only must have the force or energy *K* (see the red arrows in Fig. S2) which it needs in order to get separation at all but, just as it was hard for $C_{0}$ and $C_{1}$ to come convergence, so there is a kind of hill that $C_{0}$ and $C_{1}$ have to climb over to get separation again; they must possess not only sufficient force or energy to commence pulling apart, but also a certain surplus. It is like climbing a hill to get into a deep valley; they have to climb the hill coming in and they have to climb out of the valley the then over the hill coming back (Fig. S2). Thus, based on the above mention, we suppose that parameter *K* can be used to determines the performance of state variables, thereby influencing the disparity between forward and backward paths. Furthermore, we can also determine the total driving energy required for a complete hysteresis loop (Fig. S3).


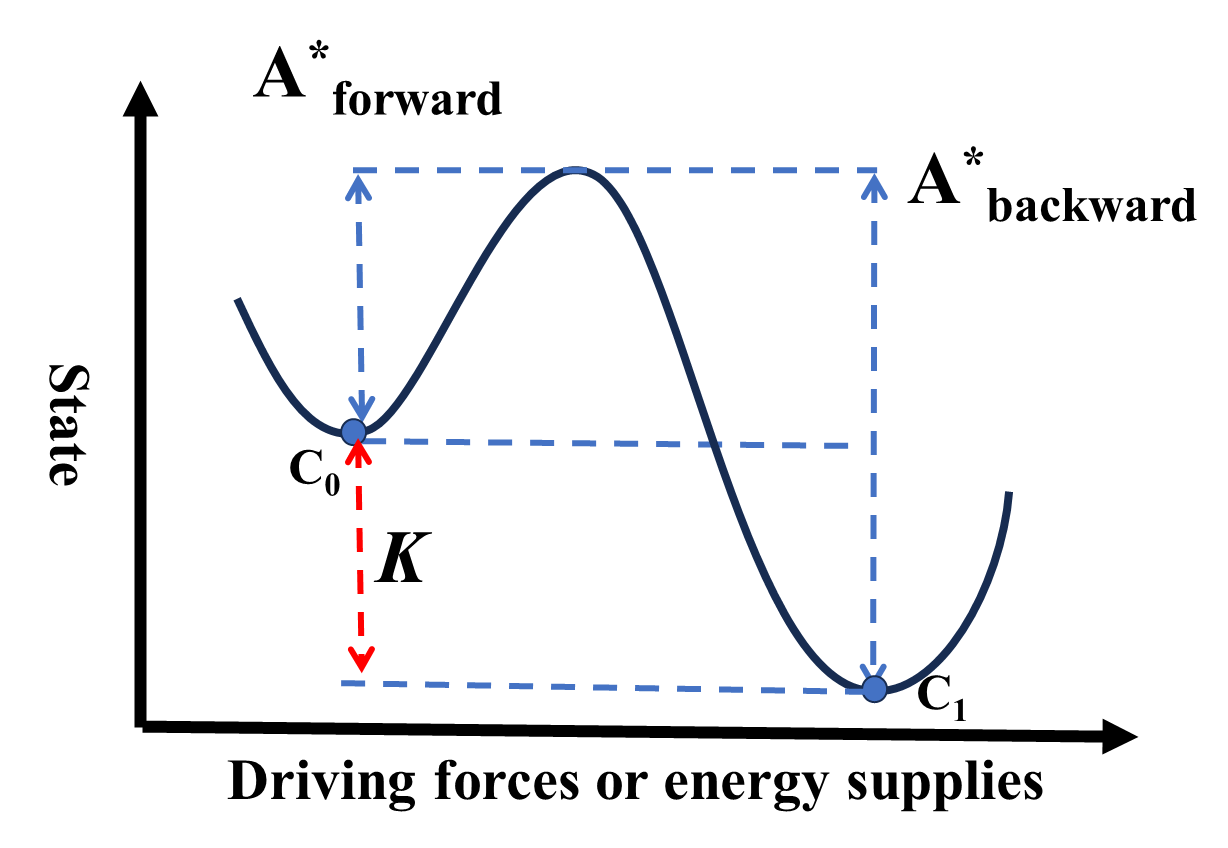


Figure S2. Thresholds of driving force or energy supply between two stable states (the initial stable state and another). A^*^_forward_ and A^*^_backward_ indicate the minimum force or energies for required for transition from to and to, respectively. The red arrow is *K* representing irreversible potential.


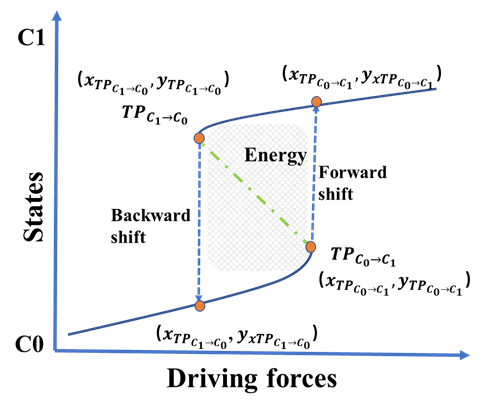


Figure S3. Schematic showing a sudden shift from the original state ($C_{0}$) to a new state ($C_{1}$) in a hysteresis system with different tipping points for forward and backward shifts. Shadow area is the required energy for driving a complete hysteresis loop. ${TP}_{C_{0}\to C_{1}}$ and ${TP}_{C_{1}\to C_{0}}$ mean the tipping points as ecosystem state transit from the original state to a new state, and return from the new state to the original state, respectively. The value in parentheses is the coordinate value corresponding to the state.

The shadowing area is calculated by next equation.

$$\begin{matrix} S=x_{C_{0}\to C_{1}}\left( y_{x_{C_{0}\to C_{1}}}-y_{C_{0}\to C_{1}} \right)-\int_{y_{C_{0}\to C_{1}}}^{y_{x_{C_{0}\to C_{1}}}} f\left( y \right)dy+\int_{y_{x_{C_{1}\to C_{0}}}}^{y_{C_{1}\to C_{0}}} f\left( y \right)dy-x_{C_{1}\to C_{0}}(y_{C_{1}\to C_{0}}-y_{x_{C_{1}\to C_{0}}}) \\ =\int_{y_{x_{C_{1}\to C_{0}}}}^{y_{C_{0}\to C_{1}}} f\left( y \right)dy-\int_{y_{C_{1}\to C_{0}}}^{y_{x_{C_{0}\to C_{1}}}} f\left( y \right)dy+x_{C_{1}\to C_{0}}\left[ \left( y_{x_{C_{0}\to C_{1}}}-y_{C_{1}\to C_{0}} \right)-\left( y_{C_{0}\to C_{1}}-y_{x_{C_{1}\to C_{0}}} \right) \right]+(x_{C_{0}\to C_{1}}-x_{C_{1}\to C_{0}})(y_{x_{C_{0}\to C_{1}}}-y_{C_{0}\to C_{1}}) \\ =\int_{y_{x_{C_{1}\to C_{0}}}}^{y_{C_{0}\to C_{1}}} (f\left( y \right)-x_{C_{1}\to C_{0}})dy-\int_{y_{C_{1}\to C_{0}}}^{y_{x_{C_{0}\to C_{1}}}} (f\left( y \right)-x_{C_{1}\to C_{0}})dy+(x_{C_{0}\to C_{1}}-x_{C_{1}\to C_{0}})(y_{x_{C_{0}\to C_{1}}}-y_{C_{0}\to C_{1}}) \\ Before hysteresis occurs, the state of the system is often very close to steady state, specifically: \\ y_{x_{C_{1}\to C_{0}}}\approx y_{C_{0}\to C_{1}}\approx C_{0},y_{C_{1}\to C_{0}}\approx y_{x_{C_{0}\to C_{1}}}\approx C_{1} \\ Therefore,\int_{y_{x_{C_{1}\to C_{0}}}}^{y_{C_{0}\to C_{1}}} (f\left( y \right)-x_{C_{1}\to C_{0}})dy- \int_{y_{C_{1}\to C_{0}}}^{y_{x_{C_{0}\to C_{1}}}} (f\left( y \right)-x_{C_{1}\to C_{0}})dy\approx0 \\ S\approx(x_{C_{0}\to C_{1}}-x_{C_{1}\to C_{0}})(y_{x_{C_{0}\to C_{1}}}-y_{C_{0}\to C_{1}})\approx\left( x_{C_{0}\to C_{1}}-x_{C_{1}\to C_{0}} \right)(C_{1}-C_{0}) \end{matrix}$$

**Analysis of the model**

The expression of our model is

$$x=f\left( Y \right)= \frac{Y-C_{0}}{C_{1}-Y}\cdot e^{\frac{K}{Y}}$$

To find a tipping-point of ecosystem transition, let’s take the derivation of the $f\left( Y \right)$

$$f^{'}\left( Y \right)=e^{\frac{K}{Y}}\frac{\left( C_{1}-C_{0}+K \right)Y^{2}-K\left( C_{1}+C_{0} \right)Y+KC_{1}C_{0}}{Y^{2}{(C_{1}-Y)}^{2}}$$

Let $f^{'}\left( Y \right)$=0, and it is equivalent to the following:

$$\left( C_{1}-C_{0}+K \right)Y^{2}-K\left( C_{1}+C_{0} \right)Y+KC_{1}C_{0}=0$$

Therefore, the existence of hysteresis is equivalent to the existence of two distinct roots of the above quadratic equation i.e.

$$\Delta={(K\left( C_{1}+C_{0} \right))}^{2}-4\left( C_{1}-C_{0}+K \right)KC_{1}C_{0}>0$$

So,

$$K>\frac{4C_{1}C_{0}}{C_{1}-C_{0}}$$

Let $K^{*}=\frac{4C_{1}C_{0}}{C_{1}-C_{0}}$, when $K>K^{*}$, tipping point is two solutions of $f^{'}\left( Y \right)=0$, that is $Y_{TPs}=\frac{K\left( C_{1}+C_{0} \right)\pm\sqrt{\Delta}}{2(C_{1}-C_{0}+K)}.$

**Standardized form for the model**

In this study, the model is expressed as:

$$x=f(Y)=\frac{Y-C_{0}}{C_{1}-Y}\cdot e^{\frac{K}{Y}}$$

This study implemented parameter standardization of the model based on three theoretical considerations:

1. **Cross-System Comparability**

Parameter normalization eliminates scale discrepancies across systems, establishing a unified benchmark. By normalizing the strength of positive feedback into dimensionless parameters, researchers can quantitatively compare hysteresis effects among systems with divergent temporal scales, spatial dimensions, and energy magnitudes. This approach is pivotal for constructing a universal theoretical framework in competitive dynamics.

Hysteresis can be observed across different ecosystems. Notable examples include the response of charophyte vegetation in shallow Lake Veluwe to changes in phosphorus concentration^1^ and state changes of oxygen in aquatic ecosystems characterized by clockwise and counterclockwise hysteresis^2^. Direct adoption of raw data extremes $C_{0}，C_{1}$ without normalization introduces parametric scale distortion. For example, we set $C_{0}=0，C_{1}=0.05$, and $C_{0}=10，C_{1}={10}^{5}$ in the sample of Ref (1) and (2), respectively. The comparison of intensity of hysteresis across different ecosystems is a challenge due to significant variations in parameters.

1. **Numerical Stability Assurance**

In nonlinear dynamic systems, non-standardized parameter combinations may induce numerical divergence during computation. As $C_{0}\to0$, the exponential term $e^{\frac{K}{Y}}$ diverges. To prevent functional divergence and ensure numerical stability, the parameter $C_{0}$​ must be restricted to a lower bound of $C_{0}\geq1$, rather than operating in the infinitesimal regime. Analogously, the parameter $C_{1}$​ requires an upper bound constraint to avoid system instability induced by unbounded amplification effects. Standardization not only prevents numerical explosions but also enhances convergence efficiency of solvers, which is particularly critical for large-scale parameter screening and long-term dynamic simulations.

1. **Preservation of Competitive Symmetry**

When competing entities undergo synchronized feedback intensity variations, the state exhibits parameter scaling invariance. Fixing positive feedback intensity as a baseline allows researchers to focus on the regulatory effects of negative feedback parameters. This dimensionality reduction strategy reveals the dominant role of negative feedback mechanisms in system transitions.

**Preprocessing method for the data**

Considering a system where one complete hysteresis loop has been recorded, we denote the measured dataset as $\left\{ \left( x_{i,1\to e}, y_{i,1\to e} \right),\left( x_{i,e\to1}, y_{i,e\to1} \right) \right\}$. When transfer is not considered, we omit the subscripts. For state data $y_{i}$, we do a linear transformation to get the standardized form of the model ($C_{0}=1$ and $C_{1}=e$) ,that is$,\varphi\left( y_{i} \right)=\frac{y_{i}-\min_{i} y_{i}}{\max_{i} y_{i}-\min_{i} y_{i}}(e-1)+1$.

. This ensures $C_{0}$ is not excessively small and $C_{1}$ not overly large. Additionally, directly scaling data into the range [1, e] via linear transformation is also feasible. which will hardly affect the experimental results. Fundamentally, the critical requirement is that the transformation constitutes a strictly monotonic bijection**.**

Subsequently, the linear transformation of $x_{i}$ is introduced. For a fixed $K'$, based on the aforementioned analysis, we can calculate the TPs of the model $\left( x_{{TP}_{1\to e}}^{m_{K^{'}}}, y_{{TP}_{1\to e}}^{m_{K^{'}}} \right)$ and $\left( x_{{TP}_{e\to1}}^{m}, y_{{TP}_{e\to1}}^{m} \right)$, The next step is to perform a linear transformation on the measured data points $\{x_{i}\}$, ${\phi_{K^{'}}(x_{i})}=\frac{x_{{TP}_{e\to1}}^{m_{K^{'}}}-x_{{TP}_{1\to e}}^{m_{K^{'}}}}{x_{{TP}_{e\to1}}-x_{{TP}_{1\to e}}}\left( x_{i}-x_{{TP}_{1\to e}} \right)+x_{{TP}_{1\to e}}^{m_{K^{'}}}$, where $x_{{TP}_{1\to e}}$ and $x_{{TP}_{e\to1}}$ are the TPs of measured data. This step ensures x-axis synchronization between the modeled TPs and the measured TPs through calibration. After analyzing the data in its standardized form, the model curve is transformed back to the original range of observed data using an inverse transformation.

Table S2 Summary of tipping points of forward (${TP}_{C_{0}\to C_{1}}$) and backward (${TP}_{C_{1}\to C_{0}}$) paths, irreversible potential (*K*), and required energy for hysteresis loop estimated by the model during state transition with hysteresis phenomenon.


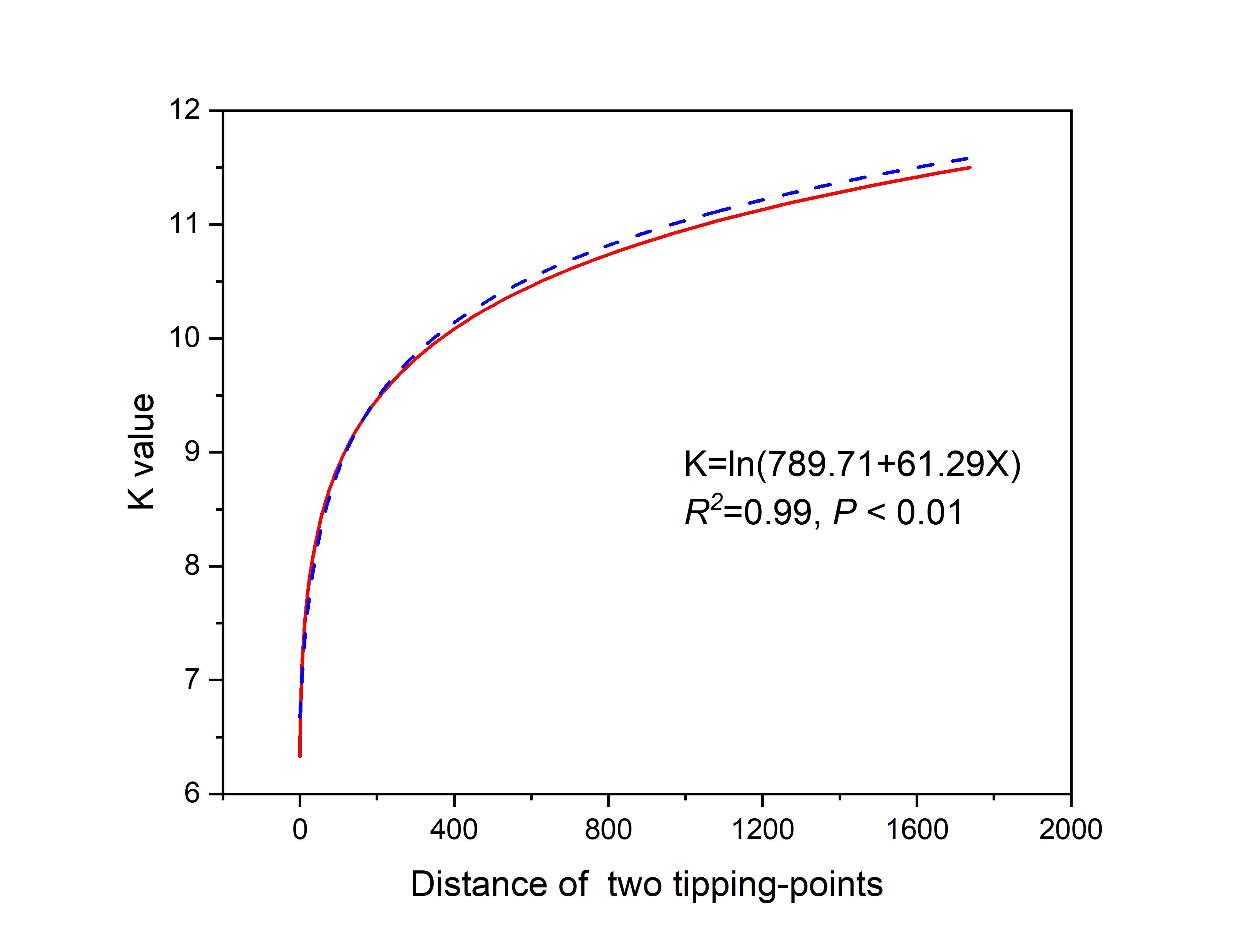


| Dataset | $\boldsymbol{TP}_{\boldsymbol{C}_{\boldsymbol{0}}\boldsymbol{\to}\boldsymbol{C}_{\boldsymbol{1}}}$ | $\boldsymbol{TP}_{\boldsymbol{C}_{\boldsymbol{1}}\boldsymbol{\to}\boldsymbol{C}_{\boldsymbol{0}}}$ | *K* | Required energy |
| --- | --- | --- | --- | --- |
| Individual leaves  （Lower BSA: mgmL^-1^） | 1.21 | 0.55 | 9.31 | 293.26 |
| Individual leaves  （medium BSA: mgmL^-1^） | 4.34 | 1.88 | 9.11 | 226.81 |
| Individual leaves  （higher BSA: mgmL^-1^） | 5.78 | 8.27 | 8.23 | 71.82 |
| Lake Veluwe  ( mgL^-1^) | 0.23 | 0.07 | 13.19 | 15478.49 |
| Grassland  ( mgNkg^-1^soil) | 1.26 | 0.45 | 9.11 | 226.81 |

Figure S4. The relationship between the irreversible potential *K* and the distance of two tipping points.

**References**

1. Meijer，M.L. Biomanipulation in the Netherlands: 15 Years of Experience.). Wageningen University (2000).
2. Northrop AC, Avalone V, Ellison AM, Ballif BA, Gotelli NJ. Clockwise and counterclockwise hysteresis characterize state changes in the same aquatic ecosystem. Ecol Lett 24, 94-101 (2021).
3. Scheffer, M., Carpenter, S. R., Foley, J. A., Folke, C. & Walker, B. Catastrophic shifts in ecosystems. Nature 413, 591-596 (2001).
